# 250. An Assessment of the Penicillin Allergy Label in Patients Undergoing Orthopedic Procedures at a VA Medical Center

**DOI:** 10.1093/ofid/ofab466.452

**Published:** 2021-12-04

**Authors:** Sherin Meledathu, MacKenzie Firek, Angelike P Liappis, Pratish C Patel

**Affiliations:** 1 Washington DC VA Medical Center, Washington, District of Columbia; 2 Belmont University College of Pharmacy, Nashville, Tennessee; 3 Washington DC Veterans Affairs Medical Center, Washington, DC

## Abstract

**Background:**

Approximately 10% of the population is labeled as penicillin (PCN) allergic, while only 1% of these individuals have a true IgE mediated allergy. This label influences the prescription of the most appropriate antibiotic and ultimately leads to antimicrobial resistance, hospital readmission, increased length of hospital stays, use of critical care beds, and greater healthcare costs. Post-surgical complications in patients undergoing total knee arthroplasty (TKA) or total hip arthroplasty (THA) are also increased when patients receive an alternative antibiotic due to PCN allergy.

**Methods:**

A retrospective chart review identified patients who underwent a TKA or THA during the 2018-2020 calendar years at the Washington DC VA Medical Center. Multiple operations at different times on the same patient were regarded as separate events. The primary outcome was patients who were evaluable for penicillin allergy de-labeling and the secondary outcome was perioperative antibiotic choice.

**Results:**

Patients in both groups were predominantly male, Black, and over the age of 60. Of a total of 317 procedures performed, we identified 28 procedures in which patients carried a PCN allergy label (PAL) and received a β-lactam alternative antibiotic for surgical prophylaxis. No patients in the PAL group received cefazolin for prophylaxis, compared to 87% of the non-PAL group who were appropriately given cefazolin. In the group carrying the PAL, 62% of patients received vancomycin and 29% of patients received clindamycin for pre-operative prophylaxis. Only one of these patients had a formal allergy consult note, but the PCN allergy was not addressed during that visit. Fewer patients (4%) required ICU admission during their hospitalization in the non-PAL group versus 10% of patients in the PAL group.

Table 1. Patient Demographics and Procedure Detail

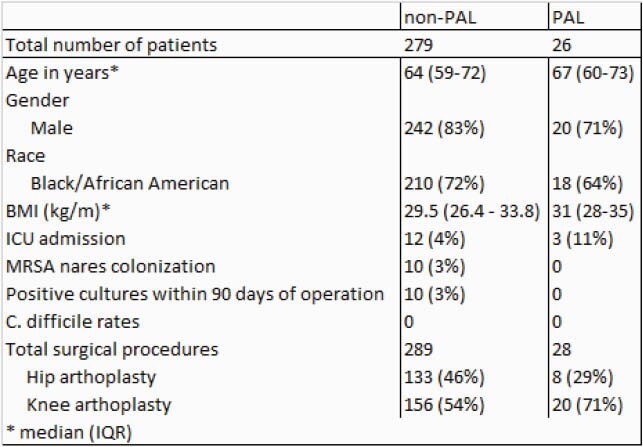

**Conclusion:**

The use of alternative antibiotics in pre-procedural prophylaxis can contribute to adverse events associated with high-risk broader spectrum antimicrobials as well as increased costs associated with antimicrobials such as vancomycin. Our facility began implementation of a penicillin de-labeling program in 2018 via skin testing and direct oral challenge in collaboration with colleagues from Allergy and Immunology. Removal of PAL in this population can increase rates of appropriate prophylaxis.

**Disclosures:**

**All Authors**: No reported disclosures

